# Supplementary material for: Booster Dose of SARS-CoV-2 mRNA Vaccine in Kidney Transplanted Patients Induces Wuhan-Hu-1 Specific Neutralizing Antibodies and T Cell Activation but Lower Response against Omicron Variant
Source: Viruses. 2023 May 9;15(5):1132. doi: 10.3390/v15051132 (PMC10224015; doi:10.3390/v15051132)
Supplement: Supplementary file 1 [file viruses-15-01132-s001.zip › Figure S1. Individual nAb response_revised.pptx]

## Slide 1
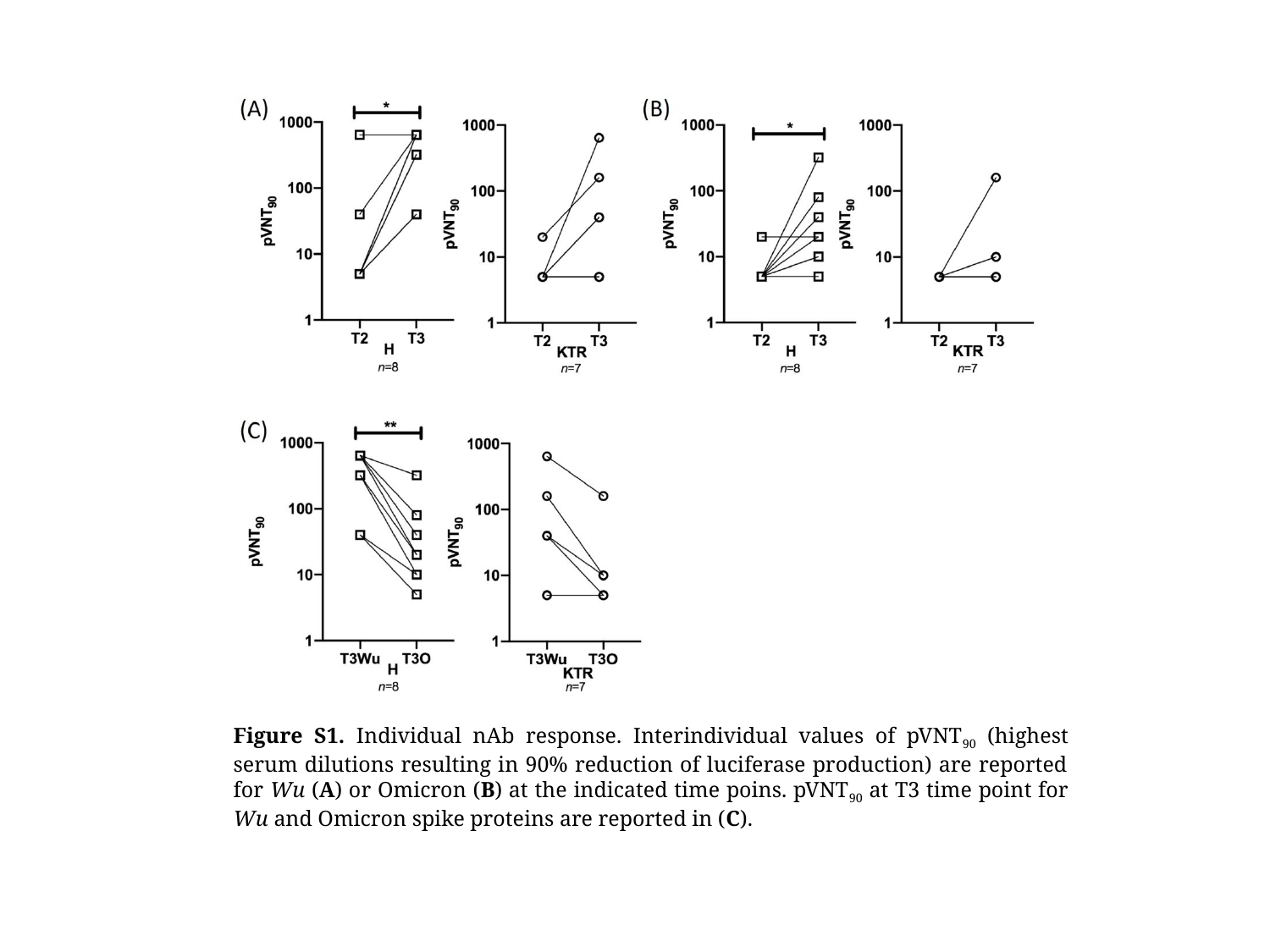

Figure S1. Individual nAb response. Interindividual values of pVNT90 (highest serum dilutions resulting in 90% reduction of luciferase production) are reported for Wu (A) or Omicron (B) at the indicated time poins. pVNT90 at T3 time point for Wu and Omicron spike proteins are reported in (C).
