# Supplementary material for: Booster Dose of SARS-CoV-2 mRNA Vaccine in Kidney Transplanted Patients Induces Wuhan-Hu-1 Specific Neutralizing Antibodies and T Cell Activation but Lower Response against Omicron Variant
Source: Viruses. 2023 May 9;15(5):1132. doi: 10.3390/v15051132 (PMC10224015; doi:10.3390/v15051132)
Supplement: Supplementary file 1 [file viruses-15-01132-s001.zip › Figure S2. T cell gating strategy.pptx]

## Slide 1
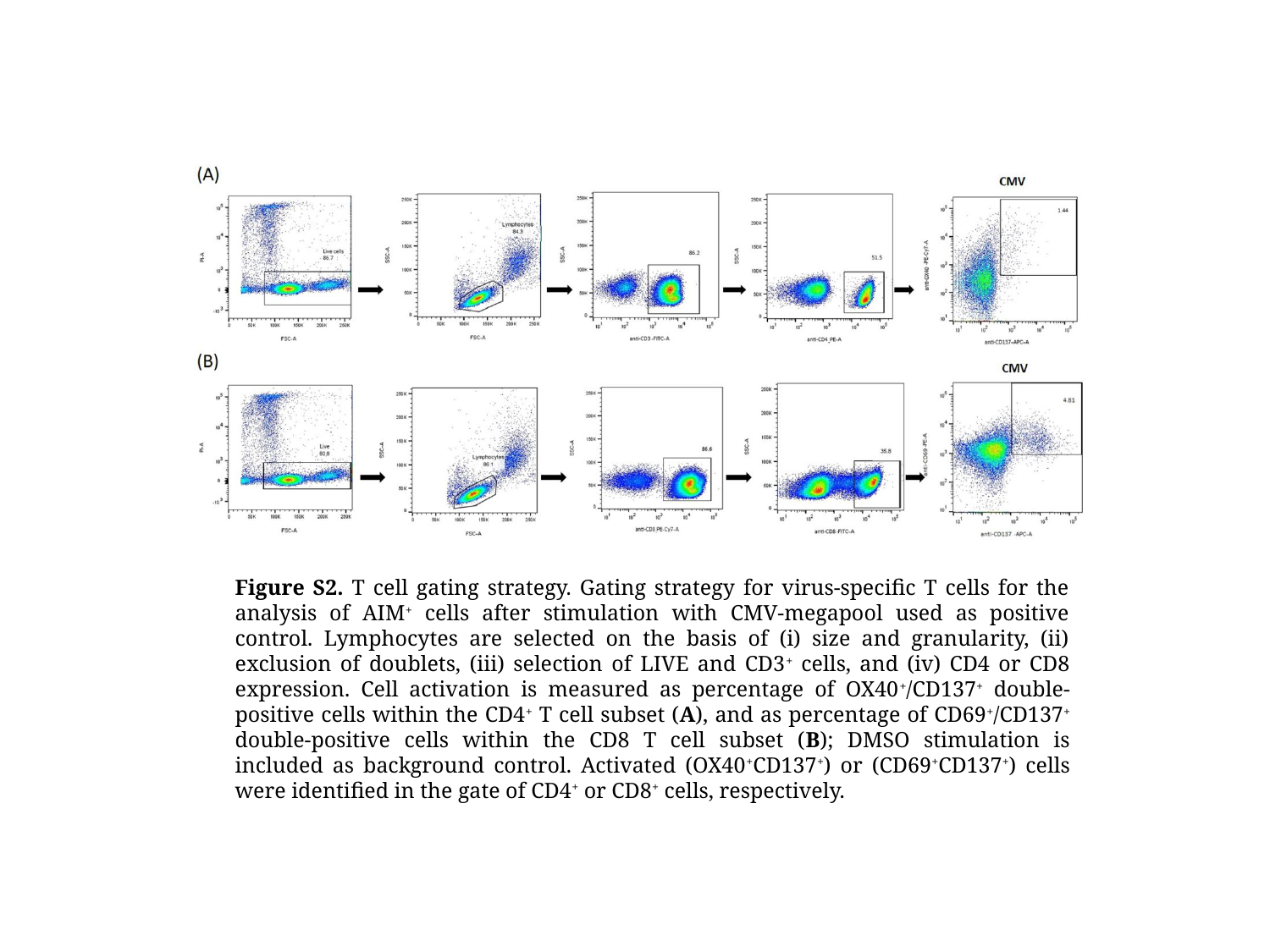

Figure S2. T cell gating strategy. Gating strategy for virus-specific T cells for the analysis of AIM+ cells after stimulation with CMV-megapool used as positive control. Lymphocytes are selected on the basis of (i) size and granularity, (ii) exclusion of doublets, (iii) selection of LIVE and CD3+ cells, and (iv) CD4 or CD8 expression. Cell activation is measured as percentage of OX40+/CD137+ double-positive cells within the CD4+ T cell subset (A), and as percentage of CD69+/CD137+ double-positive cells within the CD8 T cell subset (B); DMSO stimulation is included as background control. Activated (OX40+CD137+) or (CD69+CD137+) cells were identified in the gate of CD4+ or CD8+ cells, respectively.
