# Supplementary material for: Booster Dose of SARS-CoV-2 mRNA Vaccine in Kidney Transplanted Patients Induces Wuhan-Hu-1 Specific Neutralizing Antibodies and T Cell Activation but Lower Response against Omicron Variant
Source: Viruses. 2023 May 9;15(5):1132. doi: 10.3390/v15051132 (PMC10224015; doi:10.3390/v15051132)
Supplement: Supplementary file 1 [file viruses-15-01132-s001.zip › Figure S3. AIM representative dot plots.pptx]

## Slide 1
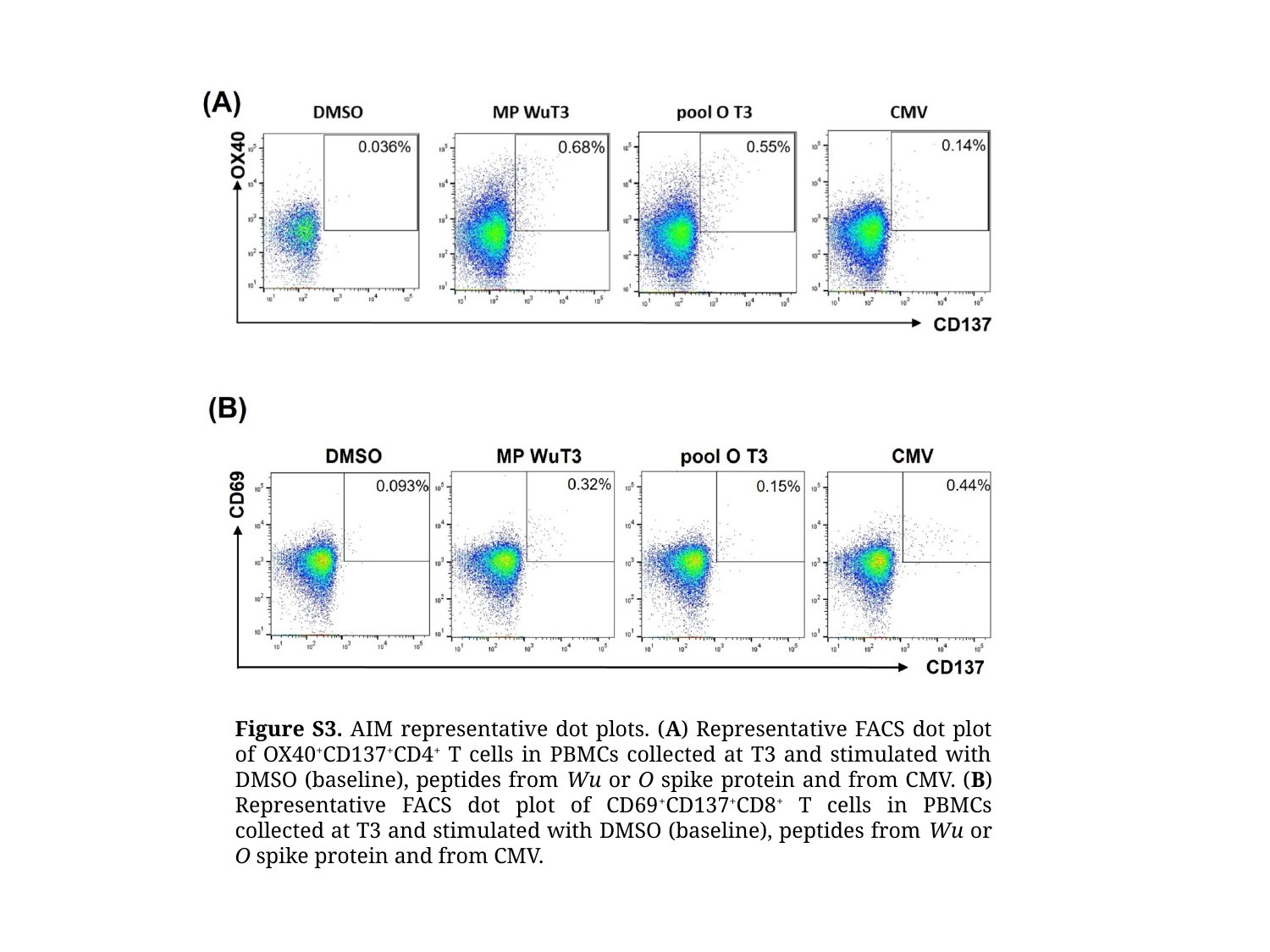

Figure S3. AIM representative dot plots. (A) Representative FACS dot plot of OX40+CD137+CD4+ T cells in PBMCs collected at T3 and stimulated with DMSO (baseline), peptides from Wu or O spike protein and from CMV. (B) Representative FACS dot plot of CD69+CD137+CD8+ T cells in PBMCs collected at T3 and stimulated with DMSO (baseline), peptides from Wu or O spike protein and from CMV.
