# Supplementary material for: Booster Dose of SARS-CoV-2 mRNA Vaccine in Kidney Transplanted Patients Induces Wuhan-Hu-1 Specific Neutralizing Antibodies and T Cell Activation but Lower Response against Omicron Variant
Source: Viruses. 2023 May 9;15(5):1132. doi: 10.3390/v15051132 (PMC10224015; doi:10.3390/v15051132)
Supplement: Supplementary file 1 [file viruses-15-01132-s001.zip › Table S1. Antibodies used for AIM assays in EVADI-COVID-19 project.docx]

| **Supplementary Table S1. Antibodies used for AIM assays in EVADI-COVID-19 project** | | | | |
| --- | --- | --- | --- | --- |
| **Antibody** | **Clone** | **Fluorochrome** | **Vendor** | **catalog number/Identifier** |
| anti-CD3 | BW264/56 | FITC | Miltenyi Biotec | 130-113-128 |
| anti-CD3 | UCHT1 | PECy7 | Biolegend | 300420/RRID: AB_439781 |
| anti-CD4 | VIT4 | PE | Miltenyi Biotec | 130-113-214/5220308514 |
| anti-CD8 | BW135/80 | FITC | Miltenyi Biotec | 130-113-157/5220308511 |
| anti-CD137 | 4B4-1 | APC | Biolegend | 309810/RRID:AB_830672 |
| anti-OX40 (CD134) | Ber-ACT35 | PE-Cy7 | Biolegend | 350012/RRID:AB_10901161a |
| anti-CD69 | FN50 | PE | BD Pharmingen | 555531/0000065498 |
| Mouse IgG1 | IS5-21F5 | APC | Miltenyi Biotec | 130-113-196/5210907921 |
| Mouse IgG | S43.10 | FITC | Miltenyi Biotec | 130-113-271 |
| Mouse IgG | S43.10 | PE | Miltenyi Biotec | 130-113-272 |
